# The path to evidence-based guidelines for food insecurity during pregnancy

**DOI:** 10.1093/eurpub/ckac129.695

**Published:** 2022-10-25

**Authors:** F McKay, P van der Pligt, J Zinga, R Lindberg, A Dickson

**Affiliations:** 1 School of Health and Social Development, Deakin University, Burwood, Australia; 2 Institute for Physical Activity and Nutrition, Deakin University, Burwood, Australia; 3 Royal Women’s Hospital, RWH, Parkville, Australia; 4 Department of Nutrition and Dietetics, Western Health, Footscray, Australia

## Abstract

**Background:**

Food insecurity has negative health implications during and after pregnancy, however, identifying and then assisting women who are food insecure is complex. Successful screening programs are often embedded in practice guidelines that include referral and treatment guidance. Screening for food insecurity is vital to address food insecurity, however, it is not present in Australia, nor are there any guidelines for healthcare settings. This presentation will describe the steps taken to gather evidence to inform the development of practice guidelines.

**Methods:**

The creation of practice guidelines for screening and responding to the needs of food insecure pregnant women was informed by 1) qualitative interviews with food insecure pregnant women, and 2) qualitative interviews with clinicians about their experiences of assisting hungry and food insecure pregnant women, 3) quantitative research with a cross-section of pregnant women about their experiences managing their food supply, 4) a systematic review describing the existing interventions addressing food insecurity during pregnancy, and 5) a modified Delphi to gather the opinions of experts on the best ways to address food insecurity in pregnancy.

**Results:**

This work highlight the potential effectiveness of a food insecurity screening tool in the antenatal setting, the readiness of clinicians to respond to this need, the breadth and depth of current interventions to address food insecurity, and the opinions of experts on how this issue needs to be addressed. The combined impact of these 5 studies is the identification of a number of responces to food insecurity and hunger during pregnancy.

**Conclusions:**

Given the lack of screening, standard care, and treatment of food insecurity in a clinical setting in Australia, it is essentail that guidelines are created that standardise patient care and control costs through efficient use of health care resources.

**Key messages:**

• Food insecurity during pregnancy has significant implications for both mother and baby.

• Creating supportive evidence-based mechanisms to address food insecurity will lead to positive outcomes.

